# Carbapenem-Resistant *Klebsiella pneumoniae* influences the outcome of early infections in liver transplant recipients

**DOI:** 10.1186/s12879-016-1876-5

**Published:** 2016-10-04

**Authors:** Francesco Barchiesi, Roberto Montalti, Pamela Castelli, Daniele Nicolini, Silvia Staffolani, Federico Mocchegiani, Alessandro Fiorentini, Esther Manso, Marco Vivarelli

**Affiliations:** 1 Clinica Malattie Infettive, Università Politecnica delle Marche, Azienda Ospedaliero-Universitaria, Ospedali Riuniti Umberto I°-Lancisi-Salesi, Via Conca, 60126 Ancona, Italy; 2 Chirurgia Epatobiliare e dei Trapianti, Università Politecnica delle Marche, Azienda Ospedaliero-Universitaria, Ospedali Riuniti Umbero I°-Lancisi-Salesi, Ancona, Italy; 3 Laboratorio di Microbiologia, Azienda Ospedaliero-Universitaria, Ospedali Riuniti Umberto I°-Lancisi-Salesi, Ancona, Italy

**Keywords:** Liver transplantation, Early infections, Antibiotic resistance, Carbapenem-resistant *Klebsiella pneumonaie*, Immunosuppression

## Abstract

**Background:**

Infections remain a leading cause of morbidity and mortality among liver transplant (LT) recipients. The aim of our study was to define the factors associated with outcome of early bacterial and fungal infections in a cohort of patients who underwent LT at the University Hospital of Ancona over a nine year period.

**Methods:**

All consecutive patients who underwent LT in our center were considered. An early infection was defined as occurring in the first month post-transplantation.

**Results:**

Among 330 patients who underwent LT from August 2005 to October 2014, 88 (27 %) had at least one infection documented within 30 days after transplantation. In 54 cases only one site was involved, in 34 cases ≥2 sites. There were 43 (30 %) pneumonia, 40 (27 %) surgical site infections, 31 (22 %) blood stream infections, and 30 (21 %) urinary tract infections. Gram-negative bacteria accounted for 64 % of the culture-positive cases, followed by Gram-positive bacteria (30 %) and fungi (6 %). A high proportion of drug-resistant strains was found within either Gram-negative (79 %) or Gram-positive (81 %) bacteria. There were 27 out 88 patients (31 %) who died within 180 days from the transplant. Factors independently associated with a higher risk of mortality were: renal replacement therapy (HR 11.797 [CI95 % 3.082–45.152], *p* < 0.0001), multisite infections (HR 4.865 [CI95 % 1.417–16.700], *p* = 0.012) and being infected with carbapenem-resistant *Klebsiella pneumoniae* (CRKP; HR 5.562 [CI95 % 1.186–26.088], *p* = 0.030).

**Conclusions:**

Overall, these data indicate that early infections in LT patients are characterized by significant mortality. In particular, an early infection caused by CRKP has an adverse impact on survival in these patients suggesting an urgent need for adopting preventive measures to avoiding this complication.

## Background

Infections remain a leading cause of morbidity and mortality among liver transplant (LT) recipients [1]. Bacterial infections are the most frequent type of infectious diseases post-transplant, followed by fungal, viral and protozoal infections [2]. A vast majority of bacterial infections occur within the first month after transplantation and most of these are caused by nosocomial organisms [3].

Accumulating data in the last several years has documented a shift towards increase in Gram-negative bacterial infections and the emergence of multi-drug-resistant (MDR) bacterial pathogens [4]. In particular, recent reports that have investigated the impact of carbapenemase-resistant- *Klebsiella pneumonia* (CRKP) in LT-recipients have documented a mortality rate up to 70 % [5]. These data underscore the importance of rigorous infection control practices to curtail the spread of resistant bacteria which are particularly difficult to manage and are associated with poor outcomes in these patients.

The aim of our study was to analyze the factors related to outcome of bacterial and fungal infections in the early post-transplant period in a cohort of patients undergoing LT.

## Methods

### Study design

This was a retrospective, observational study conducted at the Università Politecnica delle Marche, Ancona, Italy from August 2005 to October 2014. The patient population included all patients who underwent orthotopic LT and who survived more than 48 h after transplantation. The study group consisted of those patients who developed an early bacterial or fungal infection after LT. Demographic, microbiological and clinical characteristics, including preoperative, intraoperative and postoperative recipient variables, were collected and 180-day mortality from the transplant date was calculated. The present research has been performed in accordance with the ethical standards of the 1964 Declaration of Helsinki and its later amendements. The Institutional Review Board of the Azienda Ospedaliero-Universitaria Ospedali Riuniti Umberto I°-Lancisi-Salesi granted retrospective access to the data without need for individual informed consent.

### Definition and microbiology

An early infection was defined as that occurring in the first month post-transplantation. Infections were identified through active surveillance in the LT ward, and through reviews of outpatient medical records. The criteria used for defining and classifying infections were those proposed by the Centers for Disease Control and Prevention [6]. In particular, the following were considered: pneumonia, surgical site infections (SSIs, including deep intra-abdominal infections), blood stream infections (BSIs, including vascular catheter-related infections) and urinary tract infections (UTIs). Cultures for the diagnosis of bacterial or fungal infection in blood, sputum (or other respiratory secretions), urine or ascitic fluid were obtained on the basis of clinical suspicion as standard of care. Isolation of an organism from non-sterile body sites such as drainage catheters in the absence of clinical signs of infection was considered colonization. Microorganisms were cultured and identified according to standard bacteriological procedures. Susceptibility testing of the strains to antibacterial agents were performed by standard methods and the patterns were reviewed and classified according to the ESCMID (European Society for Clinical Microbiology & Infectious Diseases) guidelines [7]. In particular, MDR was defined as non-susceptibility to at least one agent in three or more antimicrobial categories; XDR (extensively drug-resistant) was defined as non-susceptibility to at least one agent in all but two or fewer antimicrobial categories; PDR (pandrug-resistant) was defined as non-susceptibility to at all agents in all antimicrobial categories.

### Statistical analysis

Patients were categorized into two subgroups based on outcome (death or survival) at 180-day from the LT. Quantitative data are depicted as median with interquartile (Q1- Q3) ranges and compared by U Mann-Whitney test. Qualitative variables were expressed as absolute and relative frequencies. Categorical variables were compared using the X^2^ test with Yates’ correction or Fisher’s exact test when appropriate. The diagnostic accuracy of selected risk factors was evaluated using receiver operating curve (ROC). We analyzed the factors associated with mortality by using a stepwise binary logistic regression model in which variables found to be significant at the univariate level (*P*-value <0.05) were introduced. Statistical analysis was performed using SPSS software, version 20 (Statistical Package for Social Sciences Inc., Chicago, IL).

## Results

Among 330 LT patients considered in the study period, 88 (27 %) had at least one infection documented within 30 days after the transplant. Demographic and clinical characteristics of these patients are reported in Table 1. Median age was 53 years, male accounted for 80 % of the population. The majority of the patients (66 %) underwent LT due to a viral infection (HCV 56 % [49/88] and HBV 15 % [13/88]). There were 9 patients HIV-coinfected. The type of anastomosis was termino-terminal in 89 % of the patients. Twenty-three percent of the patients underwent renal-replacement-therapy (RRT) in the peri-operative period.

**Table 1:** Demographic and clinical characteristics of the study cohort

Characteristics	All patients *n* = 88, (%)	180-day outcome		*p* value
		Survival *n* = 61 (%)	Death *n* = 27 (%)	
Age (years, ranges)	53 (34–67)	52 (34–66)	54 (37–67)	0.803
Gender Male	70 (80)	51 (84)	19 (70)	0.257
MELD score ≥25^a^	23 (26)	14 (23)	9 (33)	0.448
Child-Pugh stage C	42 (48)	27 (44)	15 (56)	0.436
Pre-LT hospitalization^b^	18 (20)	10 (16)	8 (30)	0.257
Previous Abdominal Surgery	7 (8)	5 (8)	2 (7)	0.900
Indication for LT - Viral^c^	58 (66)	40 (66)	18 (67)	1.000
HCV positivity	49 (56)	35 (57)	14 (52)	0.804
HBs-Ag positivity	13 (15)	8 (13)	5 (18)	0.739
HIV positivity	9 (10)	5 (8)	4 (15)	0.448
Presence of HCC^d^	30 (34)	22 (36)	8 (30)	0.731
Type of anastomosis				
Roux-en-y	10 (11)	6 (10)	4 (15)	0.488
Termino-terminal	78 (89)	55 (90)	23 (85)	
RBC units ≥ 5^e^	72 (82)	48 (79)	24 (89)	0.398
Plasma units ≥ 10	39 (44)	26 (43)	13 (48)	0.804
RRT^f^	20 (23)	5 (8)	15 (56)	<0.0001
Rejection	41 (47)	30 (49)	11 (41)	0.617
Diabetes	14 (16)	12 (20)	2 (7)	0.216
Multisite infections^g^	34 (39)	14 (23)	20 (74)	<0.0001
Pneumonia	43 (49)	22 (36)	21 (78)	0.001
SSIs^h^	40 (45)	25 (41)	15 (56)	0.301
BSIs^i^	31 (35)	14 (23)	17 (63)	0.001
UTIs^j^	30 (34)	20 (33)	10 (37)	0.885
Gram-positive bacteria	45 (51)	29 (48)	16 (59)	0.434
Gram-negative bacteria	64 (73)	40 (66)	24 (89)	0.045
Polymicrobial infections^k^	24 (27)	11 (18)	13 (48)	0.008
Fungi	9 (10)	5 (8)	4 (15)	0.448
Mixed infections^l^	6 (7)	2 (3)	4 (15)	0.069
Overall resistant infections^m^	78 (89)	52 (85)	26 (96)	0.253
CRKP infections^n^	13 (15)	4 (7)	9 (33)	0.002
CMV infection	29 (33)	14 (23)	15 (56)	0.006

^a^MELD: Model for End-Stage Liver Disease

^**b**^Pre-LT hospitalization: included any hospitalization within one month before LT

^c^Indication for LT included: viral [*n* = 58], alcoholic [*n* = 11], cryptogenetic [*n* = 7], cholestatic [*n* = 6], and other [*n* = 6] causes

^d^HCC: Hepato Cellular Carcinoma

^e^RBC: red blood cell units

^f^RRT: renal replacement therapy included: dialysis, continuous veno-venous haemo(dia)filtration and plasmapheresis

^g^Multisite infections: ≥ 2 sites (i.e.: blood and urine; blood and surgical sites etc.) were contemporarily involved

^h^SSIs: surgical sites infections

^i^BSIs: blood stream infections

^j^UTIs: urinary tract infections

^k^Polymicrobial infections: infections caused by both Gram-negative and Gram-positive bacteria

^l^Mixed infections: infections caused by both bacterial and fungal pathogens

^m^Overall resistant infections: infections caused by a bacterial pathogen showing any resistant pattern (see for details Table 3)

^n^CRKP infections: infections caused by carbapenem-resistant *Klebsiella pneumoniae* strains

In 54 out of 88 (61 %) patients only one site was involved while in 34 cases (39 %) ≥ 2 sites. A total of 144 infections were documented. There were 43 (49 %) pneumonia, 40 (45 %) SSIs, 31 (35 %) BSIs and 30 (34 %) UTIs. Gram-negative bacteria accounted for 64 % of the culture-positive cases, followed by Gram-positive bacteria (30 %) and fungi (6 %). Polymicrobial and mixed (bacterial plus fungal) infections occurred in 27 % and 7 % of the patients, respectively. Infections were due to drug-resistant bacteria in 78 (89 %) of the patients.

Pathogens isolated in the study cohort are shown in Table 2. Among Gram-negative bacteria, the most frequently isolated pathogen was *Klebsiella pneumoniae* (29 %) followed by *Pseudomonas aeruginosa* (22 %) and *Escherichia coli* (21 %) while among Gram-positive bacteria the most frequent organism was *Enterococcus faecium* (57 %) followed by *Staphylococcus aureus* (19 %) and *Staphylococcus epidermidis* (9 %).

**Table 2:** Pathogens isolated in the study cohort

Microorganisms^a^	n° (%)	Types of infection^b^				Susceptibility patterns (%)^c^
		Pneumonia	SSIs	BSIs	UTIs	
*K. pneumoniae*	33 (29)	11	9	7	6	22 CRKP (67) 2 ESBL (6) 4 MDR (12) 1 XDR (3)
						1 R (3)
						3 S (9)
*P. aeruginosa*	25 (22)	14	6	2	3	5 MDR (20) 14 R (56) 6 S (24)
*E. coli*	23 (21)	8	2	4	9	4 ESBL (17) 14 MDR (61) 5 S (22)
*S. maltophilia*	7 (6)	5	1	1	–	1 R (14) 6 S (86)
*A. baumannii*	4 (4)	3	–	1	–	1 MDR (25) 1 XDR (25) 2 R (50)
Other gram-neg.	20 (18)	4	3	6	7	5 MDR (25) 1 ESBL (5) 10 R (50) 4 S (20)
*E. faecium*	30 (57)	1	20	7	2	28 MDR (94)^d^
						1 VRE (3) 1 R (3)
*S. aureus*	10 (19)	4	5	1	–	4 MRSA (40) 6 S (60)
*S. epidermidis*	5 (9)	–	1	3	1	5 MRSE (100)
Other gram-pos.	8 (15)	3	3	–	2	6 MDR (75) 2 S (25)
*C. albicans*	4 (40)	–	1	–	3	ND
*C. glabrata*	3 (30)	–	2	1	–	ND
*C. tropicalis*	2 (20)	–	–	–	2	ND
*C. dubliniensis*	1 (10)	–	1	–	–	ND

^a^Others included: Gram negative bacteria, *Enterobacter cloacae* (n° 5), *Enterobacter aerogenes* (n° 1), *Serratia marcescens* (n°4), *Klebsiella oxytoca* (n° 2), *Acinetobacter iwoffii* (n° 2), *Acinetobacter junii* (n°1), *Haemophilus influenzae* (n° 1), *Citrobacter braaki* (n° 1), *Prevotella* spp. (n° 1), *Bacteroides uniformis* (n° 1), *Morganella morganii* (n° 1); Gram positive bacteria: *Enterococcus faecalis* (n° 2), *Streptococcus pneumonia*e (n° 2), *Staphylococcus haemolyticus* (n°2), *Staphylococcus pseudointermedius* (n° 1), *Staphylococcus* spp. (n° 1)

^b^SSIs, surgical site infections; BSIs, blood stream infections; UTIs, urinary tract infections

^c^CRKP, carbapenem-resistant *Klebsiella pneumoniae*; ESBL, extended-spectrum beta-lactamase; XDR, extensive drug resistant; MDR, multi drug resistant; MRSA, methicillin-resistant *Staphylococcus aureus*; MRSE, methicillin-resistant *Staphylococcus epidermidis*; VRE, vancomycin-resistant *Enterococcus*; R, resistance patterns not included in previous definition; S, fully susceptible; -, none; ND, not done; ^d^,there was one VRE strain within the 28 MDR

Of 112 Gram-negative bacteria, 88 isolates (79 %) were antibiotic resistant organisms and included 51 MDR and two XDR. Within the former group of isolates there were 22 carbapenem-resistant *K. pneumoniae* (CRKP) and 7 extended-spectrum beta-lactamase-producing (ESBL) bacteria. Among 53 Gram-positive bacteria, there were 43 MDR isolates (81 %), including 5 methicillin-resistant *S. epidermidis* (MRSE) and 4 methicillin-resistant *S. aureus* (MRSA). Additionally, there were 2 vancomycin-resistant *Enterococcus* (VRE) isolates one of which was also MDR. *Candida albicans* was the most commonly isolated yeasts (40 %) followed by *Candida glabrata* (30 %).

There were 27 out 88 patients (31 %) who died within 180 days from the transplant. The main cause of death was an infectious complication in 67 % of the cases, while vascular, neurological and other types of complications occurred, as main causes of death, in 19 %, 7 % and 7 % of the cases, respectively. A significantly higher proportion of patients who died within this time interval underwent RRT (Table 1). Additionally, CMV infection, pneumonia, BSIs and multisite infections were all associated with a significantly higher proportion of mortality. Similarly, infections due to Gram-negative bacteria, polymicrobial infections and infections caused by CRKP were all associated with a significantly higher proportion of mortality (Table 1).

In multivariate analysis, factors independently associated with a higher risk of mortality were: RRT (HR 11.797 [CI95 % 3.082–45.152], *p* < 0.0001), multisite infections (HR 4.865 [CI95 % 1.417–16.700], *p* = 0.012) and being infected with CRKP (HR 5.562 [CI95 % 1.186–26.088], *p* = 0.030) (Table 3).

**Table 3:** Multivariate analysis of risk factors for 180-day mortality of the study cohort

Risk factors	Hazard ratio	CI 95 %		*p* value
		Lower limit	Upper limit	
RRT^a^	11.797	3.082	45.152	<0.0001
Multisite infections^b^	4.865	1.417	16.700	0.012
CRKP infections^c^	5.562	1.186	26.088	0.030

^a^RRT: renal replacement therapy included: dialysis, continuous veno-venous haemo(dia)filtration and plasmapheresis

^b^Multisite infections: ≥ 2 sites were contemporarily involved

^c^CRKP infections: infections caused by carbapenem-resistant *Klebsiella pneumoniae* strains

## Discussion

Solid organ transplant recipients are prone to healthcare-associated infections [3]. Liver transplant recipients are especially susceptible to bacterial infections as a result of technical complexity of the surgical procedure and complications related to abdominal surgery and manipulation of the hepatobiliary system [1–3]. In this study we evaluated the factors related to outcomes of early bacterial and fungal infections in LT patients. Among 330 patients transplanted over a nine year period, 88 (27 %) had at least one infection documented within 30 days after the transplant and 27 of them (31 %) died within 180 days from the transplant. Although we investigated the contribution of as many as 30 demographic, clinical and microbiological characteristics, only three of these factors were independently associated with a higher risk of mortality. These included RRT, multisite infections, and infection due to CRKP.

Although only a minority of patients in our series (22/88) underwent RRT during the intra- and peri-operative period, this variable was significantly associated with a negative outcome. Of note, the majority of our patients undergoing this procedure were suffering from acute kidney failure which is a known risk factor for higher mortality in LT-recipients [8].

The type of infection *per se* did not influence significantly the outcome. Pneumonia and BSIs, which are generally characterized by negative outcomes in immunocompromised patients, showed to be significantly associated with high mortality rate at univariate analysis while their effect was lost in the multivariate model [9, 10]. Indeed multisite infections remained independently associated with higher risk of mortality. This can be due to the fact that involvement of two or more sites is typical of sicker patients.

As a consequence of numerous hospitalizations, invasive procedures, and frequent use of antibiotics LT, patients accumulate risk factors for infections with drug-resistant organisms [1–3]. In our series, almost 90 % of the patients suffered from early infections caused by drug-resistant bacteria. Importantly, we found that infection due to CRKP was one of the strongest predictor of post-LT mortality. This finding agrees with that recently reported by two studies showing that 1-year survival dramatically dropped from 86 % to 29 % and from 93 % to 55 % when LT patients were infected with CRKP [11, 12]. Although several therapeutic approaches have been experimented for CRKP infections including combination regimens which are usually associated with a lower risk of mortality, the management of these infections is still extremely difficult mainly in the immunocompromised host [11–15]. Overall, these data suggest that in an endemic area improved strategies for screening and prevention of CRKP infections are urgently needed. One prospective study conducted on 237 LT patients found that RRT, mechanical ventilation >48 h, HCV recurrence and colonization with CRKP at any time (i.e.: before and after LT) were all independent risk factors for CRKP infections [16]. Interestingly, based on these four variables the authors developed a risk score able to discriminate patients at low *vs* higher risk for CRKP infections [16]. Besides these essential preventive strategies, it is crucial to introduce into the market new effective antibiotics especially for those patients, such as transplant-candidates and -recipients, in which the presence of a difficult-to-treat bacterial infection may either retard a life-saving procedure or worsen an already fragile postoperative course.

The present study has several limitations. First, this was a retrospective analysis. Although we tried to collect as many clinical data as possible, we may have still missed useful information for the management of LT-patients. Second, we limited our observation to those infections occurring within one month post-OLT. This time interval was selected given that patients are at highest risk for infections during this period. Further studies addressing the prevalence of infections over a longer period of time post-OLT are ongoing in our center. Third, since our data come from a single-center experience with LT recipients belonging to an area endemic for CRKP, our findings may not be relevant to other patients populations.

## Conclusion

In conclusion, these data indicate that early infections in LT patients are characterized by significant mortality. In particular, an early infection caused by CRKP has an adverse impact on survival in these patients.

## Abbreviations

BSI: Blood stream infections
CI: Confidence interval
CMV: Cytomegalovirus
CRKP: Carbapenem-resistant *Klebsiella pneumoniae*
ESBL: Extended-spectrum beta-lactamase
ESCMID: European society for clinical microbiology & infectious diseases
HCC: Hepato cellular carcinoma
HR: Hazard ratio
LT: Liver transplantation
MDR: Multi drug-resistant
MELD: Model for end-stage liver disease
MRSA: Methicillin-resistant *S. aureus*
MRSE: Methicillin-resistant *S. epidermidis*
OLT: Orthotopic liver transplantatation
PDR: Pandrug resistant
Q1-Q3: Interquartile ranges
R: Resistant
S: Susceptible
RBC: Red blood cell units
ROC: Receiver operating curve
RRT: Renal replacement therapy
SSIs: Surgical site infections
UTIs: Urinary tract infections
VRE: Vancomycin-resistant *Enterococcus*
XDR: Extensively drug-resistant
